# Morphology Development and Mechanical Properties Variation during Cold-Drawing of Polyethylene-Clay Nanocomposite Fibers

**DOI:** 10.3390/polym9060235

**Published:** 2017-06-20

**Authors:** Bartolomeo Coppola, Paola Scarfato, Loredana Incarnato, Luciano Di Maio

**Affiliations:** Department of Industrial Engineering, University of Salerno, Via Giovanni Paolo II n. 132, 84084 Fisciano (SA), Italy; pscarfato@unisa.it (P.S.); lincarnato@unisa.it (L.I.); ldimaio@unisa.it (L.D.M.)

**Keywords:** fibers, nanocomposites, polyethylene (PE), drawing

## Abstract

In this work, the influence of composition and cold-drawing on nano- and micro-scale morphology and tensile mechanical properties of PE/organoclay nanocomposite fibers was investigated. Nanocomposites were prepared by melt compounding in a twin-screw extruder, using a maleic anhydride grafted linear low density polyethylene (LLDPE–*g*–MA) and an organomodified montmorillonite (Dellite 67G) at three different loadings (3, 5 and 10 wt %). Fibers were produced by a single-screw extruder and drawn at five draw ratios (DRs): 7.25, 10, 13.5, 16 and 19. All nanocomposites, characterized by XRD, SEM, TEM, and FT-IR techniques, showed an intercalated/exfoliated morphology. The study evidenced that the nanoclay presence significantly increases both elastic modulus (up to +115% for fibers containing 10 wt % of D67G) and drawability of as-spun nanocomposite fibers. Moreover, at fixed nanocomposite composition, the cold-drawing process increases fibers elastic modulus and tensile strength at increasing *DR*s. However, at high DRs, “face-to-edge” rearrangement phenomena of clay layers (i.e., clay layers tend to rotate and touch each other) arise in fibers at high nanoclay loadings. Finally, nanocomposite fibers show a lower diameter reduction during drawing, with respect to the plain system, and surface feature of adjustable roughness by controlling the composition and the drawing conditions.

## 1. Introduction

The use of synthetic fibers is of widespread technological importance in different fields (textiles, sport goods, military and transportation sectors, concrete reinforcement, 3D printing by Fused Deposition Modeling (FDM), etc.) and attracts extensive research efforts aimed to continuously improving their properties and processability to produce high performance fibers [1,2,3].

Among the many classes of synthetic polymer fibers, the market is dominated by polyamides, polyesters, acrylic and polyolefins. In particular, polyethylene (PE) fibers have great interest for several applications due to their easy processability, low cost, good mechanical properties, chemical resistance, lightness and resistance to abrasion. Disadvantages include the need of long chains, to provide sufficient load transfer between the weakly interacting macromolecules, low dimensional stability and low tolerance for high temperatures, which tend to cause swelling.

One possible strategy to overcome these issues, and extend the use of PE fibers to new processing technologies such as the additive manufacturing by FDM, can be the inclusion in the PE matrix of small amounts of nanosized reinforcing fillers (e.g., carbon nanotubes, organomodified layered clays, and nano-silicon dioxide). Among them, organomodified layered clays resulted particularly attractive from a technological point of view for the production of polymeric nanocomposites with unique multifunctional properties. In fact, they are widespread available and cheap, can be delaminated into nanometric clay layers with high aspect-ratio into several matrices and provide the possibility of producing nanocomposite systems with tailored and balanced strength/stiffness/toughness and improved dimensional and thermal stability, fire retardancy and gas barrier characteristics, without detrimental effects on recyclability [4,5,6,7,8,9,10,11,12,13,14,15,16]. In addition, nanoparticles can be also used as synthetic fibers coating, in order to improve mechanical properties of interfaces in composite materials [17,18,19,20,21].

Compared to the traditional filler-reinforced systems, the improved properties of polymer nanocomposites are mainly due to the stronger interfacial interaction that occurs between the polymer matrix and the silicate layers. Depending on nanocomposite morphology and clay loading, different effects on mechanical performances are reported in literature: low content and intercalated/exfoliated morphology lead to properties increase [9,22,23], while higher content and/or hybrid morphology (i.e., the presence of both tactoids and intercalated zones) produce no increase or a decrease of mechanical properties [24,25]. Moreover, the nano-platelets affect also rheological properties that are of widespread importance for process stability of polymeric systems. Generally when an intercalated/exfoliated morphology is achieved, a network structure is formed and the interactions between clay layers and polymer chains result in the improvement of both processability and drawability, due to the increase of extensional viscosity [24,26,27,28,29]. Therefore, unlike what happens for micro-composites, the use of nanofillers allows performing processes in the molten state such as fiber spinning, producing nanocomposite fibers with enhanced performances and virtually helpful in improving additive manufacturing processes.

However, remarkably for polyolefins, clay exfoliation is not easy to achieve due to the intrinsic incompatibility between hydrophilic layered silicates and hydrophobic polymers. The use of a compatibilizer, generally maleic anhydride, is thus necessary to overcome the poor compatibility between polyolefins and organoclays [12,30,31,32,33].

Even if several authors investigated the possibility of producing nanocomposite polyolefin fibers focusing on layered silicates as reinforcing filler, the combined effects of nanoclays addition and cold-drawing on the resulting morphology and fiber performances are rarely considered, particularly in compatibilized systems [24,25,34].

Within this context, we performed a study designed to investigate the effects of fiber composition and cold-drawing ratio on the evolution of the fiber nano- and micro-scale morphology (clay intercalation/exfoliation level, polymer and clay orientation, polymer crystallinity, and fiber surface defects), using a maleated PE as matrix. To this aim, PE/clay nanocomposite fibers at three different loadings of a layered organoclay (3, 5 and 10 wt %) were produced by melt compounding and extrusion, and subsequently cold-drawn at five draw ratios (7.25, 10, 13.5, 16 and 19). Morphological and mechanical properties of the nanocomposite systems were then analyzed by several techniques, i.e., X-ray diffraction (XRD), infra-red spectroscopy (FT-IR), electron and optical microscopy, and tensile mechanical testing, to evaluate the level of achieved benefits and fibers properties.

## 2. Materials and Methods

### 2.1. Materials

A linear low density polyethylene grafted with maleic anhydride, Bondyram 4108 (Polyram Industries Ltd., Ram-On, Israel), was used as matrix for the nanocomposite production. The density and the melt flow index are, respectively, 0.92 g/cm^3^ and 6.5 g/10 min (190 °C/10 kg) while the maleic anhydride (MA) content is 1 wt %.

An organomodified montmorillonite, Dellite 67G (Laviosa Chimica Mineraria S.p.A, Livorno LI, Italy), functionalized with 48 wt % of di (hydrogenated tallow)–dimethylammonium (2HT) and 115 meq/100 g of cation exchange capacity, was used as filler for composites preparation.

### 2.2. Preparation of the Nanocomposites

Prior to processing, the matrix pellets (denoted PE) and the organoclay powder (denoted D67G) were dried at 70 °C for 18 h under vacuum to remove moisture. The melt compounding was conducted using a twin-screw extruder (Collin GmbH-ZK 25, Ebersberg, Germany) with co-rotating intermeshing screws (*D*_screw_ = 25 mm, *L*/*D* = 42). A temperature profile of 180–200–200–200–200–200–200–120 °C from hopper to die was used and a screw speed of 350 rpm was set. Nanocomposite pellets (at 3, 5 and 10 wt % of silicate content) were produced by a laboratory pelletizer from the extruded strand which was quenched using a cold water bath. The samples will be called PE/xD67G where x stays for the clay percentage content. Fibers, from both matrix and nanocomposite pellets, were produced by a single screw extruder (Brabender Do-Corder E330, *D*_screw_ = 20 mm, *L*/*D* = 20, Duisburg, Germany) operating at 180, 200, and 170 °C, from hopper to die, and at 3 rpm. A capillary die (*D* = 0.5 mm, *L* = 10 mm) and a take up system with air cooling were used in order to produce undrawn filaments, with an average diameter ranging from 0.5 to 0.7 mm, which were collected on a take-up roll at 9.5 m/min.

### 2.3. Fibers Drawing

Fibers were drawn starting from spun yarns by a universal testing machine (Sans CMT6000 series, Shanghai, China) at a crosshead speed of 4 mm/min and 30 mm of gauge length. Five different draw ratios (*DR*s) were investigated, considering the ratio between the final length and the initial one (i.e., *DR* = *L_f_*/*L_o_*): 7.25, 10, 13.50, 16 and 19. Fibers drawing was performed at 25 °C.

### 2.4. Characterization

Small angle X-ray diffraction (SAXD) measurements were performed with a Bruker D8 Advance (Bruker, Billerica, MA, USA) diffractometer in the 2θ range 2–10° at a scanning rate of 0.2°/min. The interlayer distances were calculated using Bragg’s formula (Equation (1)):

*d* = *n*λ/(2 sinθ) (1)

where *n* is an integer and is equal to 1 for the first-order 001 X-ray reflection.

Wide angle X-ray diffraction (WAXD) measurements were performed using a single crystal Bruker D8 Quest diffractometer (Bruker, Billerica, MA, USA), in the 2θ range 5–55°. A single fiber was mounted on a custom-made sample holder. Two-dimensional WAXD patterns were collected using a Photon II detector (Bruker, Billerica, MA, USA) and evaluated by APEX3 software (Bruker, Billerica, MA, USA). Exposure time for each pattern was 60 s.

Both diffractometers were used operating with a Ni-filtered CuKα (λ = 0.15418 nm) radiation generated at a voltage of 35 kV and current of 40 mA.

FT-IR (Fourier Transform Infrared Spectroscopy) measurements were carried out on as-spun and drawn fibers in the range of 4000–600 cm^−1^, using a Nexus ThermoNicolet spectrometer (Waltham, MA, USA) equipped with a SmartPerformer accessory for attenuated total reflectance (ATR) analysis.

Transmission Electron Microscopy (TEM) investigations were conducted in bright field on a FEI (Hillsboro, OH, USA) Tecnai G12 Spirit-Twin (120 kV, LaB_6_) equipped with a FEI Eagle 4K CCD camera at different magnifications. Prior to observations, samples were embedded in epoxy resin and oven cured at 50 °C. Samples for TEM analysis were cut longitudinally (considering fiber axis) using an ultramicrotome Leica EM UC7 (Leica Microsystems, Wetzlar, Germany) under cryogenic conditions (nominal thickness of 100 nm), and then placed into copper grids.

As-spun and drawn fibers were observed by a Scanning Electron Microscope (SEM, mod. EVO HD 15, Carl Zeiss AG, Jena, Germany) operating at 12 kV. To observe the obtained micro-scale morphology of drawn fibers, samples were chemically etched according the procedure described by Bassett et al. [35] and widely used to verify molecular orientation and defect formation [29,36]. Fibers were immersed into a solution at 1 *w*/*v* % of potassium permanganate in a mixture of concentrated sulfuric acid, orthophosphoric acid and distilled water (10:4:1 by volume) for 2 h at room temperature. Then fibers were gold coated using an automatic sputter coater (mod. B7341, Agar Scientific, Stansted, UK) for SEM observations. Samples were cut at three different positions of as-spun and drawn fibers on three different samples, in order to ensure observations reproducibility.

Tensile properties were determined using the same machine used for cold drawing (Sans CMT6000 series, Shanghai, China) equipped with a load cell of 1 kN, according to ASTM C 1557-03. Fibers diameter was determined by an optical microscope (Zeiss Axioskop 40, Carl Zeiss AG, Jena, Germany) before the test. Tensile tests were performed at two crosshead speeds (4 and 40 mm/min) and fixed gauge length (30 mm) for determining elastic modulus (*E*) and properties at failure (tensile strength, σ_b_, and strain at failure, ε_b_), respectively.

## 3. Results

### 3.1. Morphology of As-Spun Fibers

**Figure 1:**
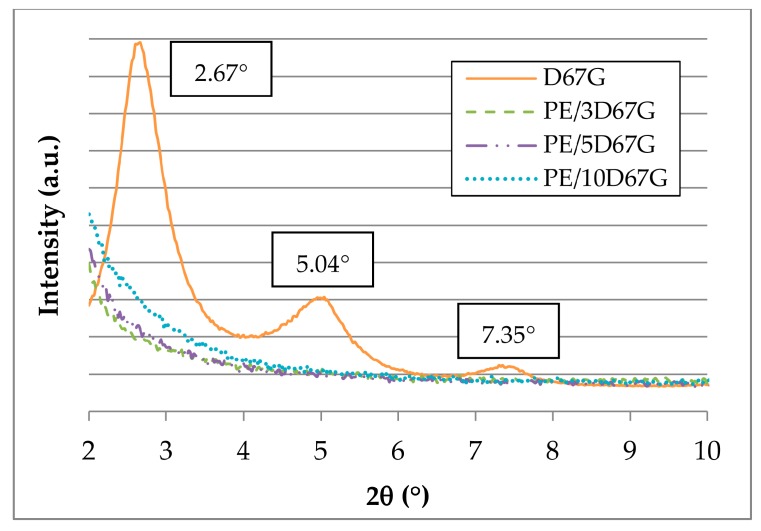
SAXD patterns of original Dellite 67G (D67G) and as-spun PE-based nanocomposite fibers at different clay content (3, 5 and 10 wt %, respectively).

Morphology of as-spun fibers was investigated by XRD, TEM and ATR-FTIR to investigate polymer/clay interactions. Figure 1 shows XRD patterns of as received D67G organoclay and as-spun PE/clay fibers at different clay loadings. The D67G pattern shows three distinct peaks at 2.67°, 5.04° and 7.35° (Figure 1). According to Bragg’s formula (Equation (1)), the three 2θ values reported in Figure 1 correspond at the following interlayer distances: 3.3, 1.8 and 1.2 nm, respectively. The first peak, corresponding at the (001) reflection, is representative of the regularly stacked layers; the high order reflections, corresponding to the (002) and (003) reflections, derive from the regular arrangement of the intercalant [37]. Considering PE/clay fibers, it can be seen that in every case the three sharp peaks of D67G disappeared, indicating the achievement of intercalated/exfoliated structure with interlayer higher than 4.4 nm (corresponding at 2θ value < 2°).

**Figure 2:**
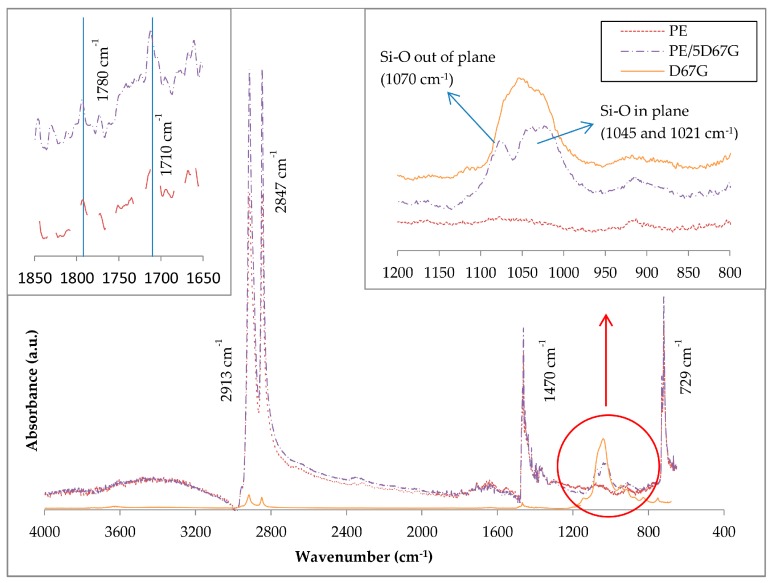
ATR-FTIR spectra of Dellite 67G, PE and PE/5D67G as-spun fibers in the range 600–4000 cm^−1^. Detail of the regions 1650–1850 cm^−1^ and 800–1200 cm^−1^ are also reported.

The organoclay delamination was verified also by ATR-FTIR that allows relating the increase of the dispersion level of the clay to the change in its Si–O bond absorption band [38]. As demonstrated for several natural and organomodified clays (montmorillonite, bentonite and hectorite), Si–O bond appears as one broad bond when the material is agglomerated and is resolved in two peaks (in-plane and out-of-plane) after delamination. The ATR-FTIR spectra for D67G, PE and PE/5D67G as-spun fibers are compared in Figure 2. The main peak assignments are listed in Table 1 [39]. The PE sample shows all the characteristic peaks of polyethylene grafted with maleic anhydride (MA). The appearance of the carbonyl stretching bands and the ring stretching vibrations of saturated cyclic five-membered anhydride confirms that MA is grafted onto PE. The D67G sample shows one broad Si–O band in the 1150–950 cm^−1^ region, where clays typically absorb IR radiation, and two low peaks in the 2950–2800 cm^−1^ region, corresponding to CH_2_ stretching due to the organic modifier. The PE/5D67G sample shows all the signals of its two components but in its spectrum. The Si–O signal is resolved in two absorption bands: the out-of-plane at 1070 cm^−1^ and the in-plane band at 1045 and 1021 cm^−1^, as a consequence of the clay layer delamination occurred during melt compounding.

**Figure 3:**
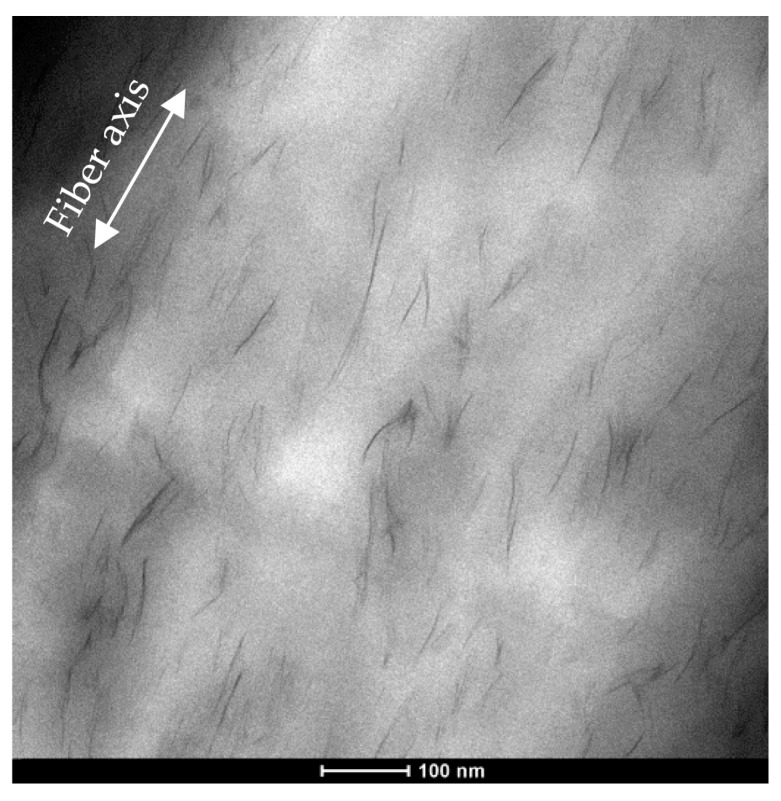
TEM image of as-spun PE/5D67G fiber (section parallel to fiber axis).

To better understand the obtained morphology in terms of clay distribution and orientation, TEM investigations were carried out on undrawn fibers, on sections parallel to the fiber axis. As representative case, a micrograph of longitudinal section of PE/5D67G as-spun fiber is reported in Figure 3. In addition, TEM confirms the high intercalation/exfoliation level of the layered silicate in the PE matrix and evidences that as-spun fibers display silicate layers partially oriented along flow direction, as a consequence of the shear deformation associated to the flow inside the extruder, as reported in literature on other nanocomposite systems [28,40,41,42].

**Figure 4:**
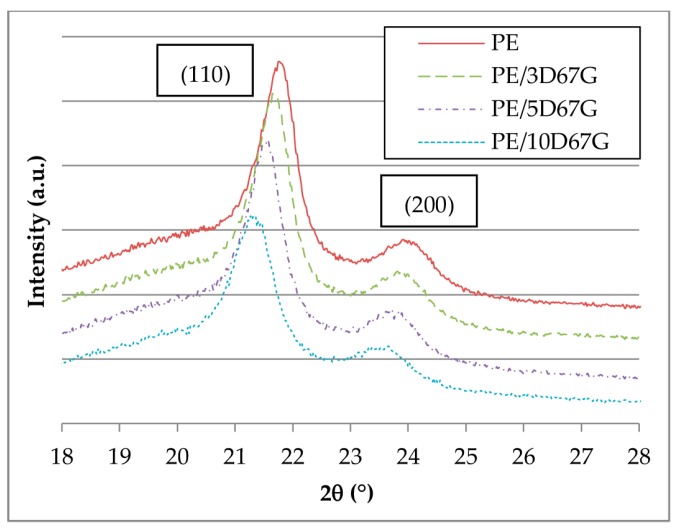
WAXD patterns of nanocomposite fibers.

Wide angle X-ray diffraction (WAXD) patterns report the presence of the typical crystalline structure of PE with two dominant peaks at 2θ angles of 21.6° and 23.9°, which correspond to the 110 plane and the 200 plane of an orthorhombic crystal structure (Figure 4), respectively. At increasing nanoclay content, a considerable decrease in the intensity and a slight broadening and shift towards lower 2θ of the 110 reflection is observed while a lower influence was registered for the 200 reflection. According to the literature, the presence of an intercalated/exfoliated morphology, combined to the high interfacial area and adhesion between the polymer and the clay, hinders PE chain mobility, having two effects: a reduction of the crystallinity degree (related to the peak intensity) and dimension of the crystallites (related to the peak broadening and shift) [23,25,43].

### 3.2. Mechanical Properties of As-Spun Fibers

**Figure 5:**
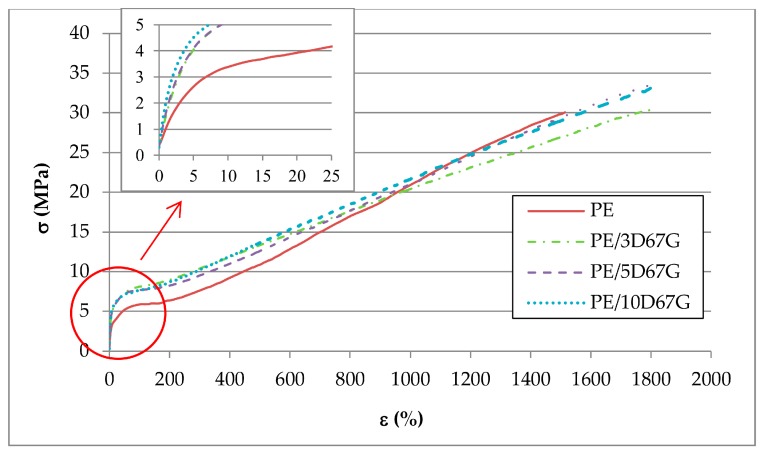
Tensile stress (σ)/strain (ε) curves of as-spun PE, PE/3D67G, PE/5D67G and PE/10D67G fibers.

To investigate the influence of nanoclay addition on mechanical performances of as-spun fibers, tensile tests were performed. The effect of clay amount on the tensile stress–strain curves of as-spun fibers is shown in Figure 5.

Nanocomposite fibers exhibit not only a considerable increase of the elastic modulus but also a significant increase of ductility. In fact, as-spun PE fibers show a lower strain at failure (about 1500%) compared to nanocomposite fibers. Even if, unfortunately, the maximum strain of the apparatus (i.e., 1800%, corresponding to 540 mm of elongation) did not lead to failure of nanocomposite fibers, hence a conclusive analysis of their cold-drawability is not possible, and nanoclay addition significantly postpones fiber failure. Similar toughening effect was described in literature by other authors for several polymer/clay nanocomposite systems with intercalated/exfoliated morphology and was explained as a consequence of temporary polymer chains physical crosslinking created by the nanoclay and resulting in local regions of enhanced strength, so that the growth of microdefects is retarded [29].

Moreover, with respect to the neat PE fibers, the nanocomposite ones show also a lower slope of their stress/strain curves after yielding, which implicates a reduction of the polymer chains orientation during the tensile test due to hindering effect of nanoclays [23,24,25,43].

**Figure 6:**
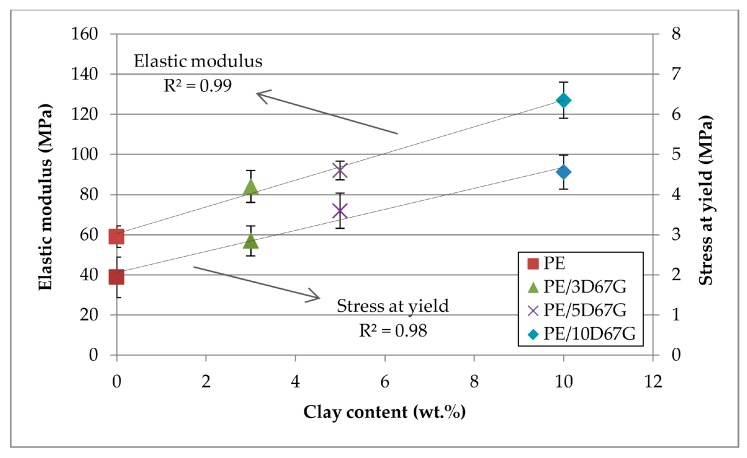
As-spun fibers elastic modulus and stress at yield correlated to the nanoclay wt % (0, 3, 5 and 10, respectively).

Elastic modulus of undrawn fibers at different nanoclay contents is reported in Figure 6. The influence of clay layers on mechanical properties was significant, corresponding to an increase of the elastic modulus of 42%, 56% and 115% for 3, 5 and 10 wt % of nanoclay, respectively. Moreover, the presence of a linear relationship between elastic modulus and clay content is clearly recognizable. The same behavior was observed also for stress at yield (Figure 6).

### 3.3. Structural and Morphological Characterization of Drawn Fibers

**Figure 7:**
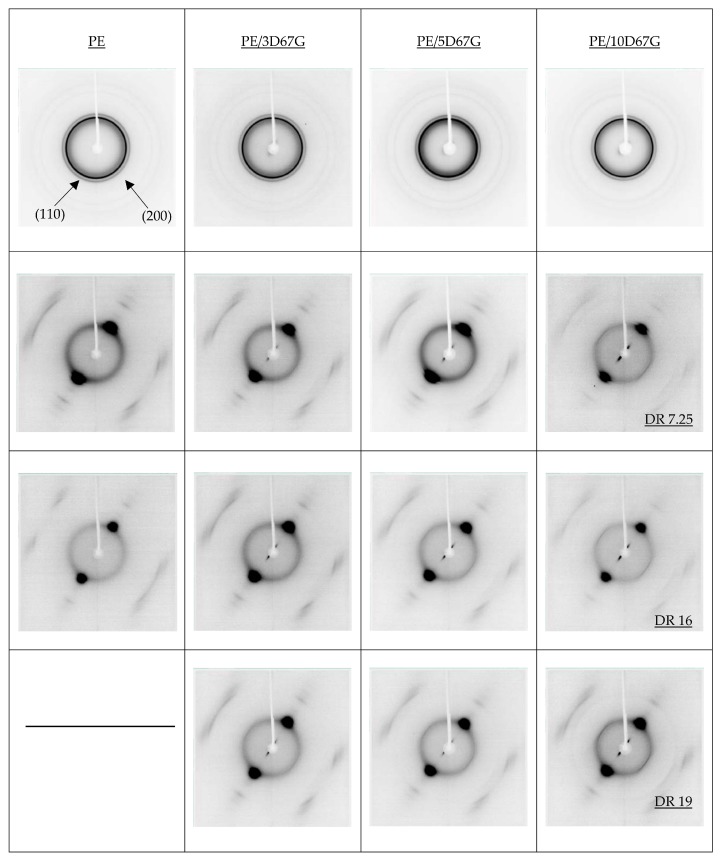
WAXD patterns for the investigated composite fibers at the different DRs (as-spun, 7.25, 16 and 19, respectively).

The structure and morphology of nanocomposite fibers after cold cold-drawing were studied by XRD, FT-IR and microscopy investigations. WAXD patterns of as spun and drawn fibers, at different *DR*s, are reported in Figure 7. The fiber axis, indicated by an arrow in Figure 7, corresponds to the meridian direction. The diffraction patterns of as spun samples show two sharp rings corresponding to orthorhombic lattice planes (200) and (110) of PE, which are non-oriented in all cases, superimposed on the amorphous halo.

No clay reflections can be observed, as expected, because the nanocomposite low diffraction angles are out of the measurement range (5°–55°). On the contrary, after drawing, strong reflections appear, at small angles (near the beam stopper) along equatorial lines for the nanocomposite fibers. These reflections, more intense at increasing clay content, suggest the morphology modification related to orientation and to distance reduction of the of silicate layers during the cold drawing process, as also found in other polymer/layered clay nanocomposite fibers [28,42,44,45]. With concern to the matrix morphology, even at the lowest *DR* = 7.25, XRD patterns show the presence of well oriented crystallites and poorly oriented material, related to the formation of oriented semi-crystalline fibers (Figure 7). At higher DRs, only a slight increase of orientation is observed, as a consequence of a two-slip process (interlamellar and intralamellar deformations) as described in the literature [38,46,47].

**Figure 8:**
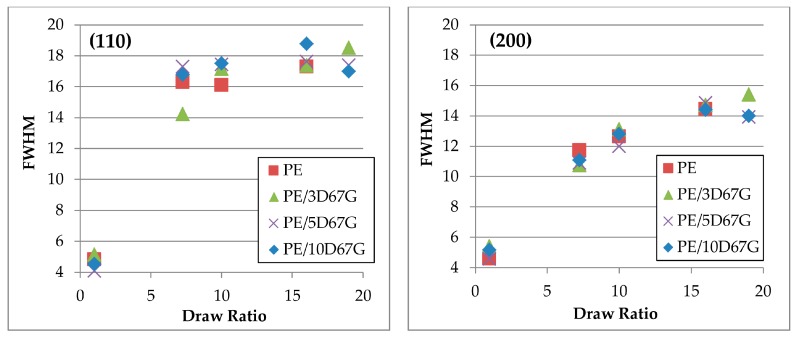
Changes of FWHM in equatorial (110) and (200) reflection against draw ratio of the investigated fibers.

To analyze in more depth the crystallographic deformation with strain, full width at half-maximum (FWHM) values were determined along the equatorial line (Figure 8) for the orthorhombic lattice planes (110) and (200). According to Sherrer’s formula, at increasing FWHM, the average thickness of crystallites of polyethylene decreases. As evident, the increase of FWHM in (110) reflections is prevalent than that of (200) reflections. Such behavior can be explained by the chain slip in polyethylene crystals, resulting in fibrils formation, that occurs preferably in the (110) planes that present weak van der Waals interactions rather than in the (200) planes that have more strong covalent bonds [47,48].

Coherently to what previously observed from WAXD patterns (Figure 7), FWHM values of PE/5D67G and PE/10D67G show a maximum for a *DR* of 16. On the contrary, PE/3D67G shows a progressive increase of FWHM at increasing DRs, corresponding to a lower thickness of crystallites.

**Figure 9:**
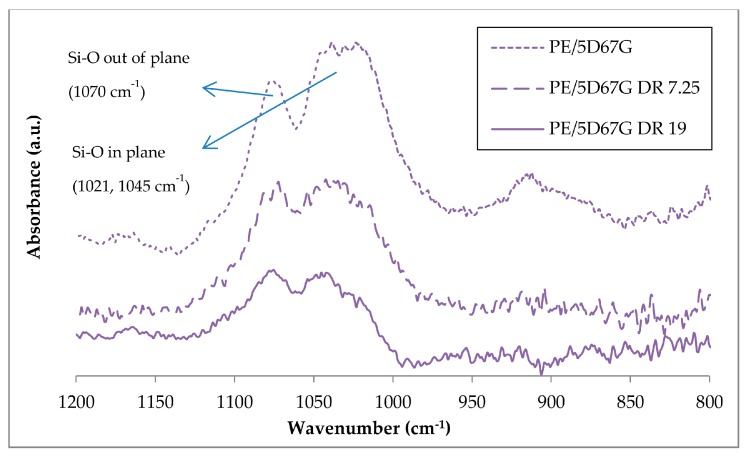
ATR-FTIR of PE/5D67G fibers at different draw ratios (i.e., as-spun, 7.25 and 19) in the range 1200–800 cm^−1^.

**Figure 10:**
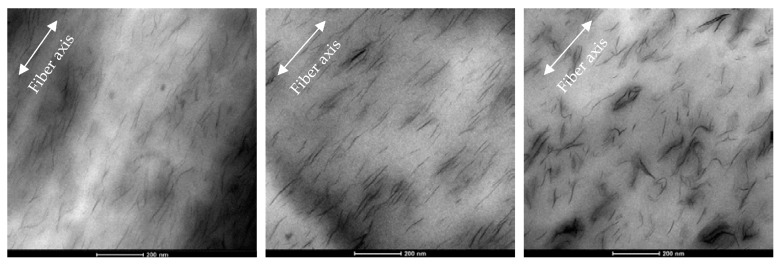
TEM images of longitudinal sections of PE/5D67G fibers: as-spun (**left**); drawn at *DR* 7.25 (**center**); and at *DR* 19 (**right**), respectively.

The nanoclay morphology evolution related to cold drawing process was investigated by ATR-FTIR and TEM analyses, as shown in Figure 9 and Figure 10, respectively, for PE/5D67G fibers at different *DR*s. The ATR-FTIR spectra in Figure 9 indicate the occurrence of clay layers rearrangement phenomena with a reduction of the clay layer delamination level, as it can be inferred by the intensity decrease and the tendency to the merging of the Si–O signals, at increasing DRs, particularly relevant for the in-plane absorption bands (i.e., at 1045 and 1021 cm^−1^).

The TEM images in Figure 10 demonstrate that, with respect to the as-spun system, at low *DR*s, there is an improvement of clay orientation along the fiber axis (i.e., the extrusion direction), while, at high *DR*s, there is the occurrence of self-assembling phenomena of the clay layers, which tend to rotate and touch each other, due to electrostatic attractions between positively charged edges and negatively charged faces, as described in the literature [28,42,49]. Such a “face-to-edge” morphology is made possible during cold-drawing process, which gradually reduces fibers diameter, by the good adhesion between clay layers and polymer matrix achieved using a compatibilized PE matrix.

**Figure 11:**
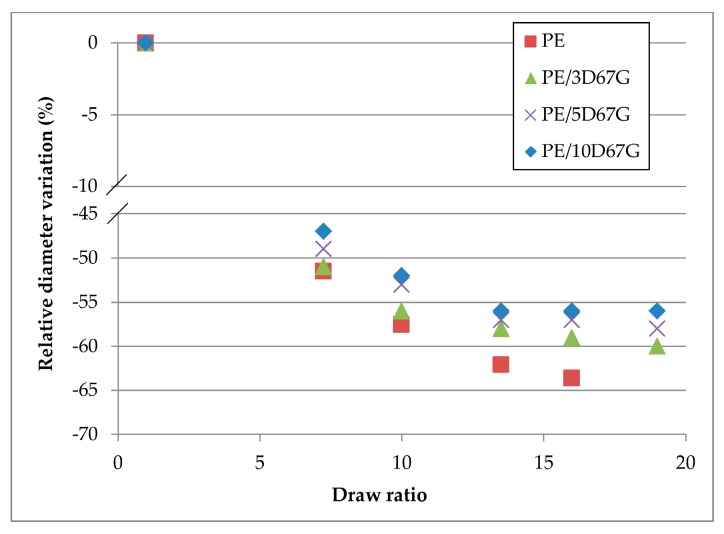
Relative diameter variation after drawing for the investigated fibers (i.e., PE, PE/3D67G, PE/5D67G and PE/10D67G).

Optical microscopy allowed to investigate the changes, at increasing DRs and clay wt %, in the fiber macroscale morphology in terms of diameter reduction. The relative diameter variations (Δ*d*/*d*_0_, %) for all the investigated fibers at each DR are compared in Figure 11. The graph shows a general reduction of the fiber diameter during cold-drawing: the decrease is continuous for PE and PE/3D67G systems, whereas reaches a plateau at DR 13.5 for PE/5D67G and PE/10D67G ones. Moreover, with respect to the neat PE fibers, the relative diameter variation is progressively lower for nanocomposite fibers at increasing nanoclay loading. Again, the phenomenon is related to the clay particles that act as a rigid body inside the PE matrix hindering the polymer chains mobility and reducing their alignment during stretching.

**Figure 12:**
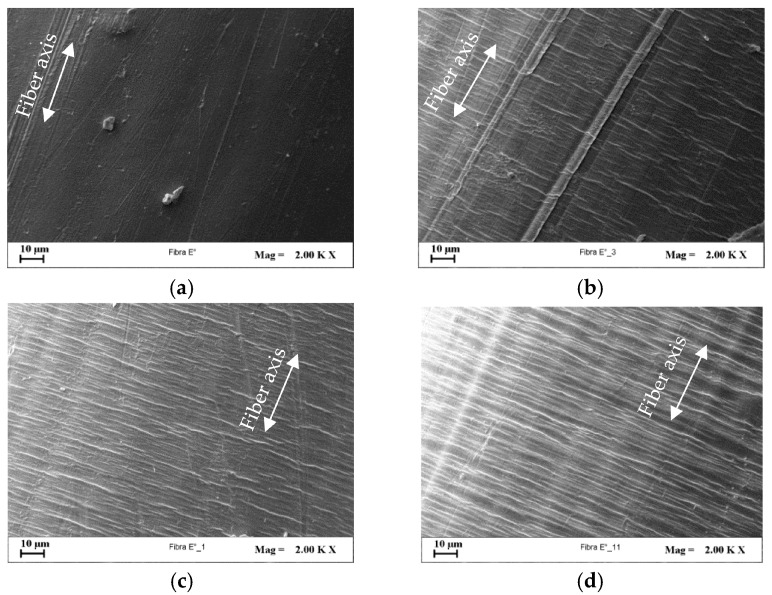
SEM micrographs (2000×) of PE/3D67G fibers surface: (**a**) as-spun fiber; (**b**) *DR* = 7.25; (**c**) *DR* = 13.50; and (**d**) *DR* = 19.

SEM investigations were performed to detect the effects of cold-drawing and nanoclay on the development of microscopic fiber surface defects. The fiber surface patterning, in fact, plays a key role on mechanical performance of fiber reinforced composite systems since it strongly affects the fiber wettability and the fiber/matrix interfacial adhesion, which generally increase at increasing fiber roughness for many polymer systems, even if also the surface chemistry contributes to regulate the whole behavior [50]. Since all the investigated systems at increasing *DR* showed similar occurrence of defect formation, the PE/3D67G was chosen as example. Figure 12 shows that the fiber surface is quite smooth in undrawn system and increases its roughness during drawing. In particular, at intermediate *DR*s (Figure 12b,c), transversal band appearance and growing are recognizable; at the highest *DR*, the transversal rows extend across the whole width of the fiber (Figure 12d). Such defects occur in systems having low polymer chains mobility and thus low capability of the macromolecules to be extended and aligned during drawing [29,35,51,52,53,54].

**Figure 13:**
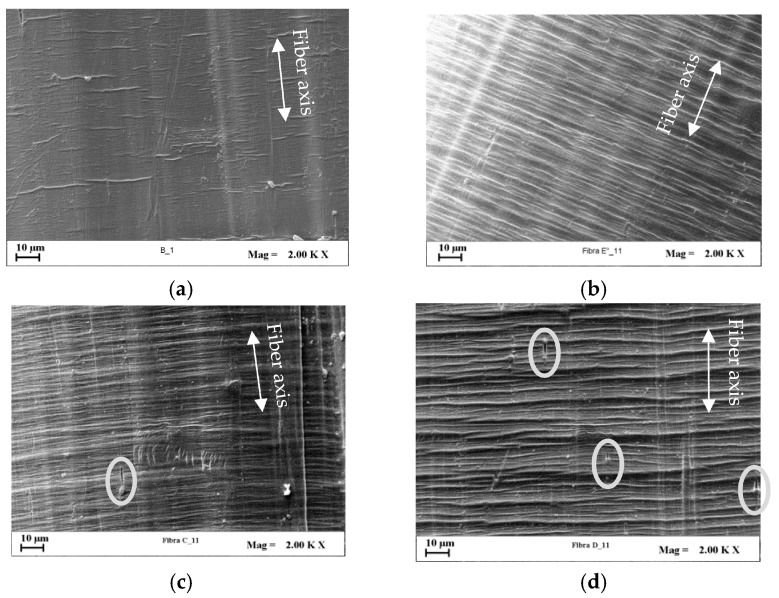
SEM micrographs (2000×) of fibers surface at their respective highest *DR*: (**a**) PE at *DR* 16; (**b**) PE/3D67G; (**c**) PE/5D67G; and (**d**) PE/10D67G, at *DR* 19.

Moreover, the presence of cavities on fibers surface can be recognized (Figure 13c,d). This kind of superficial defect is also well-known for traditional micro-composites and has to be related to the formation, during fiber drawing, of clay agglomerates that act as stress concentrators promoting the voids setup [48,55,56]. In addition, at increasing nanoclay content, the surface roughness is more pronounced.

**Figure 14:**
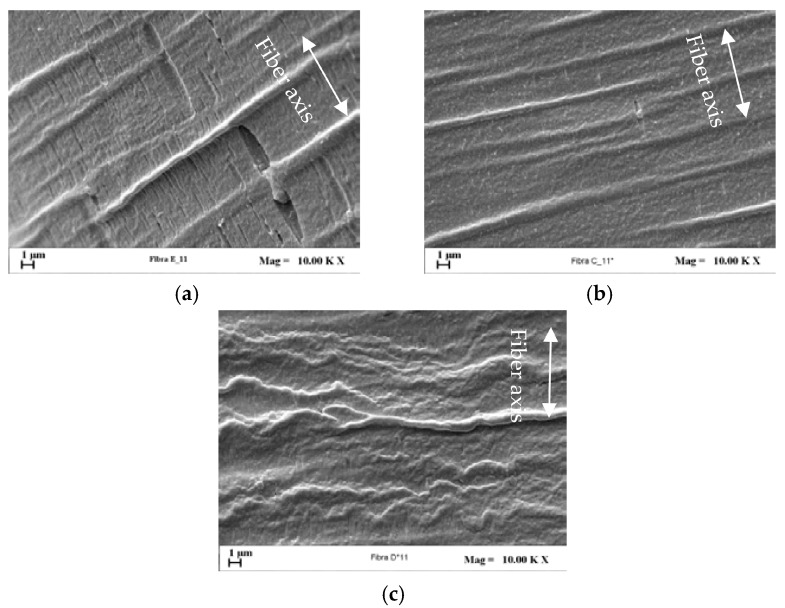
SEM micrographs (10000×) of etched fibers: (**a**) PE/3D67G; (**b**) PE/5D67G; and (**c**) PE/10D67G, at *DR* 19 (white arrow indicates the fiber axis).

To analyze more in depth fibers surface topology, after fiber etching SEM images were taken to recognize in the samples zones having different density of the polymer matrix. Figure 14 compares the micrographs obtained on nanocomposite fibers drawn at the maximum investigated *DR* (i.e., 19). The PE/3D67G fiber shows a morphology denoted in the literature as “Pisa structure” [35] and due to voids opening due to the etchant and occurring in low density zones. The density deficiency of these regions results from the macromolecular rearrangements related to the material transformation into a fibrillar structure. This morphology is not present in the PE/5D67G and PE/10D67G systems, which indicates a more homogenous density distribution and thus a delayed transition towards a fibrillar structure, as a consequence of the higher amount of nanoclay in these systems that reduces the polymer chains mobility. These observations suggest that, at the considered DR, with respect to the PE/3D67G systems, the PE/5D67G and PE/10D67G fibers are more far from the fiber rupture limit resulting in their improved drawability.

### 3.4. Mechanical Properties of Drawn Fibers

**Figure 15:**
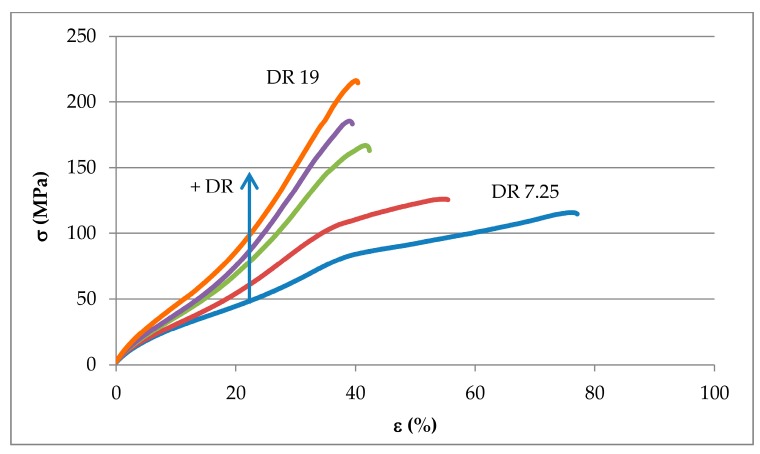
Stress/strain curve for PE/3D67G drawn fibers at different *DR*s (7.25, 10, 13.5, 16 and 19).

The effects of cold-drawing and nanoclay content on fibers mechanical performance were assessed carrying out tensile tests. Nanoclays addition increases fibers ductility and nanocomposite fibers reached the maximum achievable value of crosshead displacement. For all nanocomposite systems, at increasing *DR*, higher tensile strengths are achieved while a ductility reduction was observed, as shown in Figure 15 for the PE/3D67G fibers, as a consequence of defect formation that initiates premature tensile failure. Moreover, the fibers drawn at *DR* equal to 16 and 19 have approximately the same strain at failure, as expected considering that they have also similar diameter after drawing (Figure 11).

**Figure 16:**
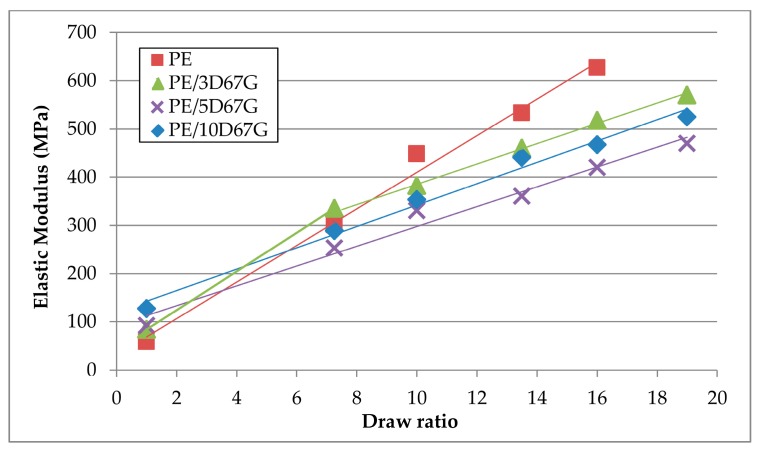
Increase of the elastic modulus at increasing draw ratios (*DR*s) of PE, PE/3D67G, PE/5D67G and PE/10D67G fibers, standard deviation ±10%.

Fiber cold-drawing produces an overall increase of mechanical properties at increasing *DR*s, as shown in Figure 16. The elastic modulus of neat PE increases more than that of nanocomposites fibers. This behavior was expected since nanocomposite fibers at higher *DR*s show higher diameters with respect to neat PE fibers, confirming the nanoclay hindering effect.

**Figure 17:**
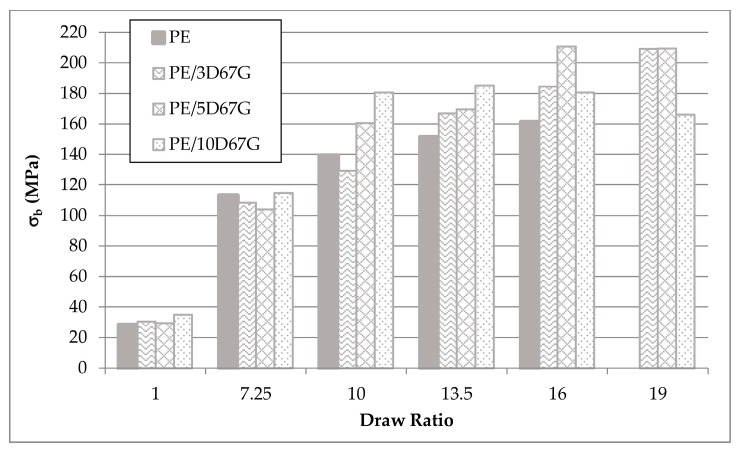
Tensile strength (σ_b_) of PE, PE/3D67G, PE/5D67G and PE/10D67G drawn fibers at the investigated *DR*s (7.25, 10, 13.5, 16 and 19), standard deviation ±5%.

However, in fact, nanocomposite fibers always have better performances, in terms of tensile strength, compared to PE fibers and significant differences between the blends are recognizable for draw ratios higher than 10 (Figure 17). PE and PE/3D67G fibers exhibit a progressive increase of tensile strength with *DR*; on the contrary, PE/5D67G and PE/10D67G show a maximum in the tensile strength values at *DR*s 16 and 13.50, respectively. This behavior is coherent with the structural variations previously observed, in terms of crystallite thickness and orientation and defect formation.

## 4. Conclusions

The results obtained in this work allowed analyzing the relationships among composition, cold-drawing extent, morphology evolution and mechanical performance improvements in PE/clay nanocomposite fibers.

All the as-spun fibers showed intercalated/exfoliated morphology and partial orientation of the nanoclay along the fiber axis even before drawing. This resulted in beneficial effects on the mechanical behavior of the as-spun fibers in terms of elastic modulus, which increases up to 115% for the more loaded system, and drawability, thanks to the efficient level of stress transfer between the polymer matrix and the nano-dispersed clay platelets.

Morphological XRD and microscopy investigations evidenced that, in general, the cold-drawing process promotes the polymer chain orientation along the drawing direction. For neat PE and PE/3D67G fibers, the matrix orientation increases continuously with the *DR*. On the contrary, for PE/5D67G and PE/10D67G, a maximum level of orientation is obtained at intermediate *DR* due to hindering effect of the nanoclay on polymer chain mobility. Regarding the nanoclay, after an initial increase of the silicate layer orientation at low *DR*s, the cold-drawing determines the occurrence of silicate rearrangement phenomena with the development of a “face-to-edge” morphology, which was related to the good adhesion between clay layers and polymer matrix. These clay rearrangements reduce the polymer chains mobility and are responsible of the development of the surface micro-defects and of the delayed transition towards a fibrillar structure in more loaded systems (i.e., PE/5D67G and PE/10D67G), as inferred by SEM investigations.

Because of the matrix orientation and clay rearrangements, a relevant increase of the tensile strength (up to ca. 600% for PE/3D67G and PE/5D67G), drawability and dimensional stability were obtained.

The straightforward approach to combining organomodified layered silicate with maleated PE showed interesting performance benefits helpful for enhancement of additive manufacturing technologies such as Fused Deposition Modeling and other rapid prototyping methods.

## Figures and Tables

**Table 1:** Absorption bands and their peak assignments.

Bands (cm^−1^)	Assignments
3600–3200	–OH stretching vibration
2913	–CH_2_ asymmetric stretching
2847	–CH_2_ symmetric stretching
1790	C=O symmetric stretching
1710	C=O symmetric stretching
1470	–CH_2_ bending deformation
1070	Si–O out of plane bending
1045, 1021	Si–O in plane bending
919	Ring stretching vibration of saturated cyclic anhydride
729	–CH_2_ rocking vibration
